# Supplementary material for: Validation of the Polish version of the Motivational Postures (Toward Taxes) Questionnaire
Source: PLoS One. 2021 Jun 15;16(6):e0252937. doi: 10.1371/journal.pone.0252937 (PMC8205165; doi:10.1371/journal.pone.0252937)
Supplement: S1 File — (PDF) [file pone.0252937.s001.pdf]

System podatkowy i Urząd Skarbowy w Polsce (Braithwaite, adaptacja: Kołodziej)

Poniżej znajdują się stwierdzenia, które opisują stosunek ludzi do systemu podatkowego i Urzędu Skarbowego. Otocz kółkiem cyfrę najbliższą Twojej opinii.

1 – Zupełnie się nie zgadzam

2 – Nie zgadzam się

3 – Nie mam zdania

4 – Zgadzam się

5 – Zupełnie się zgadzam

1. Płacenie podatków jest słuszne	1	2	3	4	5
2. Sumienne wypełnianie obowiązków podatkowych najprawdopodobniej zaowocuje dobrymi relacjami z Urzędem Skarbowym	1	2	3	4	5
3. Nawet jeśli stwierdzę, że postępuję niezgodnie z wymaganiami Urzędu Skarbowego, nie będę się z tego powodu zamartwiać	1	2	3	4	5
4. Wywiązanie się ze wszystkich obowiązków względem Urzędu Skarbowego nie jest możliwe	1	2	3	4	5
5. System podatkowy nie jest idealny, ale działa wystarczająco dobrze dla większości z nas	1	2	3	4	5
6. Płacenie podatków to moralny obowiązek	1	2	3	4	5
7. Jeśli Urząd Skarbowy zastosuje wobec mnie jakieś restrykcje, wówczas przestanę wypełniać moje obowiązki podatkowe	1	2	3	4	5
8. Płacenie podatków jest według mnie formą pomocy rządowi w realizacji wartościowych celów	1	2	3	4	5

9. Jeśli trudności z regulowaniem zobowiązań podatkowych nie są zawinione przez podatnika, Urząd Skarbowy będzie do niego pozytywnie nastawiony	1	2	3	4	5
10. Urząd Skarbowy nie powinien narzucać swojej woli podatnikom	1	2	3	4	5
11. Akceptuję odpowiedzialność związaną ze sprawiedliwym płaceniem moich podatków	1	2	3	4	5
12. Lubię rozmawiać ze znajomymi o istniejących w systemie podatkowym lukach prawnych	1	2	3	4	5
13. Płacenie podatków wynika z poczucia odpowiedzialności i powinno być akceptowane przez wszystkich podatników	1	2	3	4	5
14. Przyznanie się przed Urzędem Skarbowym do popełnionego w zeznaniu podatkowym błędu jest korzystne	1	2	3	4	5
15. Płacenie podatków jest korzystne dla wszystkich	1	2	3	4	5
16. Lubię szukać luk w prawie podatkowym	1	2	3	4	5
17. Gdy raz Urząd Skarbowy zaliczy Cię do grupy nieposłusznych podatników, to nigdy już nie zmieni zdania	1	2	3	4	5
18. Właściwie nie wiem, czego ode mnie oczekuje Urząd Skarbowy i nie zamierzam się o to pytać	1	2	3	4	5
19. Więcej osób powinno się sprzeciwiać Urzędowi Skarbowemu	1	2	3	4	5
20. Nie interesuje mnie to, czy według Urzędu Skarbowego postępuję właściwie	1	2	3	4	5
