# Supplementary material for: Validation of the Polish version of the Motivational Postures (Toward Taxes) Questionnaire
Source: PLoS One. 2021 Jun 15;16(6):e0252937. doi: 10.1371/journal.pone.0252937 (PMC8205165; doi:10.1371/journal.pone.0252937)
Supplement: Attachment — Submitted filename: Response to Reviewers.docx [file pone.0252937.s002.docx]

Dear Frantisek Sudzina,

Academic Editor

Thank you very much for the letter and insightful reviews. I have read them carefully and made corrections in line with those suggestions. I hope this work improved the manuscript.

Below, please find the detailed information about changes made in the manuscript.

1. With reference to the suggestions formulated by Reviewer #1:

- The issue of person and contextual characteristics that shape motivational postures was underlined in the Discussion section.
- Description of correlations between Individual and Social Tax Standards Scale and MPQ was corrected in order to clarify the text (line 509-510).
- In line with the suggestion the assumption regarding relations between Pleasant Game scale and belief in life as a zero-sum game was corrected (line 515). I agree that gameplaying in “Defiance in Taxation and Governance” clearly shows game playing as a Zero-sum game life view.

2. With reference to the suggestions formulated by Reviewer #2:

- I agree that in case of scales with small (less than 10) number of questions item-rest of the scale correlations are often included. However, in this paper in order to ensure the comparability with the original scale I decided to present the same indicator as in the original work. It should be noted that both versions of the scale (Australian and Polish) consist of similar (below 10) number of items. Therefore, I decided not to change the reliability measure.

3. Manuscript meets PLOS ONE's style requirements, including those for file naming.
4. Abstract – citations were removed from the abstract. Therefore, the reference list order was changed. All papers remained on the reference list.
5. Additional details regarding participants consent were provided in Participants sections in all 3 studies.
6. All typos were corrected.

I am looking forward to hearing from you.

Sincerely,

Sabina Kołodziej
